# The Effect of Low-Temperature Short-Term Annealing on the Microstructure and Properties of Ultrafine-Grained Pure Titanium

**DOI:** 10.3390/ma18030517

**Published:** 2025-01-23

**Authors:** Yanxia Gu, Jinghua Jiang, Aibin Ma, Yaxiao Gu, Zhenquan Yang, Haoran Wu, Chenlong Song, Qingping Yang, Chaobing Ni

**Affiliations:** 1 College of Marine and Electrical Engineering, Jiangsu Maritime Institute, Nanjing 211100, China; 2 College of Materials Science and Engineering, Hohai University, Nanjing 210098, China; aibin-ma@hhu.edu.cn (A.M.); 230208020001@hhu.edu.cn (Y.G.); yang-zhen-quan@hhu.edu.cn (Z.Y.); 231625010026@hhu.edu.cn (C.S.); 231625010039@hhu.edu.cn (Q.Y.); 231625010021@hhu.edu.cn (C.N.); 3 Suqian Institute, Hohai University, Suqian 223800, China; 4 Institute of Materials, Henan Academy of Sciences, Zhengzhou 450046, China; haoranwuted@hnas.ac.cn

**Keywords:** titanium, annealing, texture strengthening

## Abstract

Industrial pure titanium was processed through 1–4 passes by equal-channel angular pressing (ECAP), and the processed samples were subsequently short-term annealed for 15 min at 300 °C, to achieve better mechanical properties for industrial applications. The microstructure was analyzed using TEM, EBSD, and XRD observations. The mechanical properties were studied through tensile testing. The TEM and EBSD results showed that the grain size of industrial pure titanium was refined to approximately 420 nm after four passes of ECAP processing, with very little grain growth after annealing. The XRD analysis proved the enhanced basal texture in the subsequent annealed samples. Tensile tests indicated that the strength of the processed sample increased with more ECAP passes and was improved by 39% after four passes compared with the as-received state; in addition, the low-temperature short-term annealing resulted in a further strengthening phenomenon. It was concluded that the strengthening after annealing in industrial pure titanium was likely due to the improved basal texture, resulting in texture strengthening.

## 1. Introduction

In recent years, ultrafine-grained (UFG) materials have received much attention owing to their remarkable mechanical properties, and extensive research has been carried out on the associated preparation methods, microstructure observation, and properties of ultrafine crystalline materials [1,2]. The implementation of severe plastic deformation (SPD) methods has facilitated the fabrication of ultrafine crystalline microstructures [3,4,5]. Among various deformation methods, equal-channel angular pressing (ECAP) is a potential method for SPD, which enables the production of ultrafine crystalline bulk metals with uniform microstructures and large dimensions [6,7]. The sample is forced through two channels with an identical cross-section in the die, which are orientated at an angle relative to each other. This process induces shear deformation at the channel corners, resulting in substantial grain refinement. Titanium alloys are characterized by the light weight nature, high strength, low elasticity, and desirable corrosion resistance and have considerable potential in various fields, including aerospace, marine engineering, and biomedicine [8,9,10,11,12]. Existing research has demonstrated that the mechanical properties [13,14,15,16,17,18] and corrosion resistance [19,20,21] of titanium alloys can be further enhanced via ECAP, which considerably broadens the potential applications of these materials and meets the high demands [22,23]. In dental surgery, the Ti-6Al-4V alloy is often used as a medical implant due to its higher strength compared to pure Ti. However, the Al and V atoms are harmful to human health. It was reported that the strength of UFG pure Ti processed by SPD was comparable to that of Ti-6Al-4V, indicating its potential for use in long-term dental implants [11,24,25,26].

Although SPD exhibits considerable advantages, the ultrafine crystalline materials tend to exhibit unstable microstructures owing to the accumulation of numerous dislocations and internal stresses, which can adversely affect their performance and reliability in subsequent applications [27,28]. To address this issue, appropriate annealing heat treatment can be used. Several researchers have reported that reasonable annealing of ultrafine-grained materials could result in the further improvement of their strength, ductility, electrical conductivity, and surface properties [29,30,31]. Mavlyutov et al. [29] demonstrated that the annealing of UFG Al at 90–200 °C led to an increase in microhardness and electrical conductivity. Orlova et al. [30] reported that annealing a UFG Al-Zr alloy at 230 °C for 60 min led to an increase in strength and electrical conductivity simultaneously, which was attributed to the rearrangement of grain boundaries. Kim et al. [32] reported that annealing at 300 °C for 60 min brought about an increased strength in nanoscale high Mn steel, which was associated with C clusters driven by annealing. Chen et al. [33] demonstrated that the strength of a Cu-Zn alloy was further enhanced by annealing at 300 °C for 30 min. Sotniczuk et al. [31,34] found that annealing UFG pure Ti at 250 °C for 15 min led to an enhancement of microhardness and corrosion resistance. Majchrowicz et al. [35] reported that a strength enhancement of UFG pure Ti was obtained by annealing at 25 °C for 15 min. The above unexpected phenomenon is primarily attributed to the recovery of the ultrafine structure [35,36], or the segregation of impurity atoms [37]. Because texture evolution occurs during annealing [38], texture likely plays a significant role in enhancing mechanical properties and corrosion resistance. Although several studies have been carried out on the preparation methods and properties of ultrafine crystalline materials, comparatively fewer studies have explored the influences of annealing on the microstructure and mechanical properties of these materials. In particular, the role of texture in the strength enhancement of UFG materials after annealing has not been comprehensively investigated.

In this research, the effect of low-temperature short-term annealing treatment on industrial pure titanium processed via ECAP for 1–4 passes was examined. The modified microstructures and properties of the specimens after each pass were systematically discussed to investigate further improvements in the properties of pure titanium subjected to ECAP.

## 2. Materials and Methods

**Figure 1:**
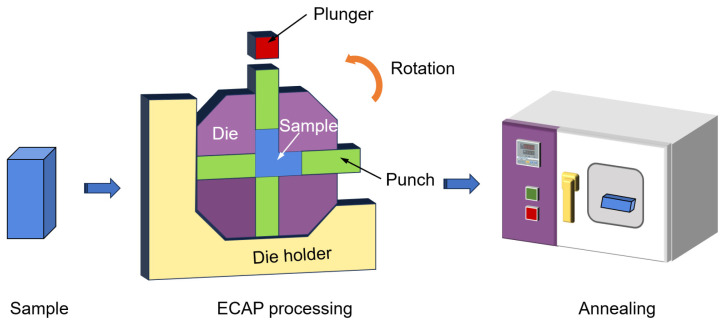
The schematic of the rotary-die ECAP and annealing treatment.

Experiments were performed using industrial pure titanium (TA2, Baimtec Material Co., Ltd., Beijing, China); the chemical composition of this material is provided in Table 1. The initial grain size was ~10 μm. The samples subjected to ECAP (Wuxi Haofei Machinery Factory, Wuxi, China) were sectioned into dimensions of 20 mm × 20 mm × 45 mm. As illustrated in Figure 1, the samples were processed through a rotary die at a temperature of 420 °C for 1–4 passes. There were two orthogonal channels in the die. The right, left, and bottom punches were loaded in the channels, and then the sample and the top punch were placed into the die. The plunger pressed the sample out of the right channel. It was unnecessary to remove the sample from the die during the pressing passes; instead, the die was simply rotated anticlockwise to facilitate the subsequent pass—the detailed operation steps were described in our early work [7]. After ECAP processing, the samples underwent annealing at 300 °C for 15 min, and the mechanical and microstructurally tested specimens were selected from the longitudinal section of the sample. For simplicity, the samples investigated after these procedures are summarized in Table 2.

For room-temperature tensile tests, the spacing dimensions of each specimen were 6 mm × 2 mm × 2 mm (length × width × height). The tests were conducted at a temperature and tensile rate of 25 °C and 1 mm/min, respectively. Each test was performed in triplicate to ensure satisfactory data reproducibility and accuracy.

Transmission electron microscopy (TEM, FEI Tecnai G T20, Hillsboro, OR, USA) foils were prepared for microstructure analysis by mechanically grinding them to 0.1 mm, followed by electrolytic double-jet thinning using a perchloric acid–alcohol mixture as the electrolyte. The crystalline structures were analyzed using a X-ray diffractometer (D8 Discover, Bruker, Billerica, MA, USA) with Cu Kα radiation from 30° to 80°.

Electron Backscattered Diffraction (EBSD) tests of samples were performed using a scanning electron microscope (SEM, Gemini 300, Carl Zeiss, Oberkochen, Germany) equipped with an Oxford EBSD detector. The working voltage was at 20 kV. The specimens had dimensions of 10 mm × 5 mm × 2 mm. The specimens were grinded using metallographic sandpapers, and electrochemically polished in the mixed solution of perchloric acid and methanol (1:9 by volume). The electrolytic polishing was performed at −30 °C with a voltage of 30 V and an electric current of 0.8 A. The scanning area was 57 μm × 42 μm and the step size was 0.09 μm for the 2P and 2P + A samples. The scanning area was 30 μm × 20 μm and the step size was 0.045 μm for the 4P and 4P + A samples. The kernel average misorientation (KAM) mapping was calculated according to the following formula: KAM=∑(∆φi)/n, where ∆φi represents the grain boundary angle of each grain boundary, and *n* represents the total number of grain boundaries.

## 3. Results and Discussion

### 3.1. Microstructure

**Figure 2:**
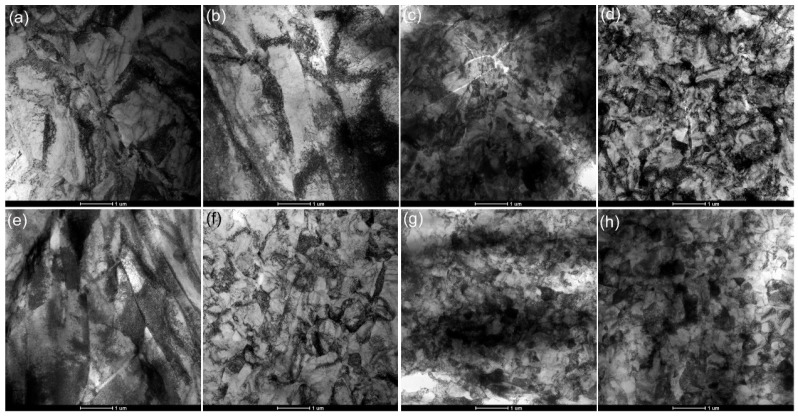
TEM images of pure titanium processed via various ECAP passes and annealing: (**a**) 1P; (**b**) 2P; (**c**) 3P; (**d**) 4P; (**e**) 1P + A; (**f**) 2P + A; (**g**) 3P + A; (**h**) 4P + A.

Figure 2 displays the TEM microstructure after each ECAP pass and subsequent low-temperature short-term annealing treatment. The deformation-twinning structure and considerably coarse undeformed grain boundaries can be identified, as illustrated in Figure 2a. Furthermore, the microstructure exhibits pronounced dislocation entanglement, but clear sub-grain boundaries have not been formed, indicating that the structure remains predominantly deformed after a single pass. After two passes (Figure 2b), numerous dislocation tangle zones are distributed within the material and coarse slats with uneven widths are visible. As shown in Figure 2c, an increased number of pressing passes generates enhanced grain deformation, accompanied by dislocation annihilation or rearrangement, resulting in the gradual formation of small dislocation cells. As illustrated in Figure 2d, the dislocation walls forming the cell wall can continuously absorb surrounding dislocations to form sub-grain boundaries. This process results in more distinct grain boundaries and the development of finer grains with an average crystallite size of ~420 nm. The grain size was similar to the previous work of pure Ti after ECAP reported by Stolyarov et al. [39]. During annealing, it was reported that the grain growth of UFG pure Ti started at 350 °C and higher [40]. Valiev et al. [40] found that the annealing strengthening effect was optimum at a temperature range of 250–300 °C, and there was little annealing strengthening effect below 250 °C. Therefore, this research focused on the microstructure after annealing at 300 °C. Our previous work found that annealing at 300 °C with a duration of 30 min or longer caused visible grain growth and a decrease in strength [41]. So, the microstructure evolution of each-pass samples after heating at 300 °C for 15 min was investigated in this work. As shown in Figure 2e–h, when the subsequent annealing treatment was applied after various pressing passes, minimal growth in grain size was observed due to low annealing temperatures and short annealing duration [42]. Furthermore, the recovery process reduced the dislocation density and led to the development of distinct grain boundaries.

**Figure 3:**
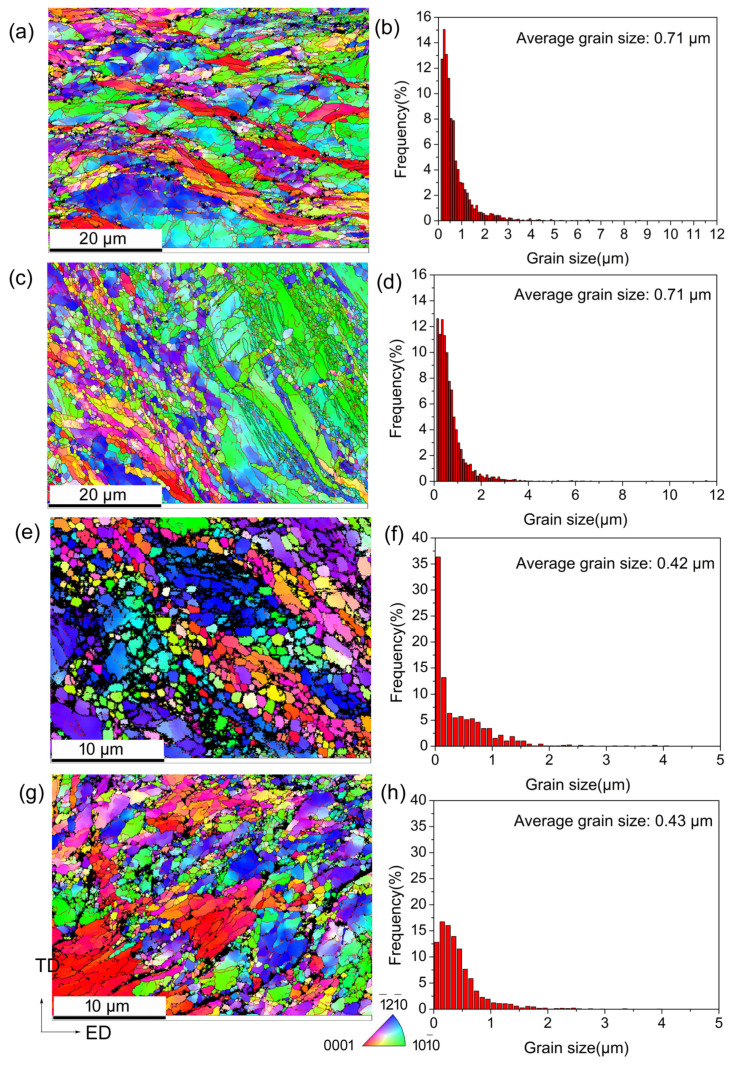
EBSD IPF maps (**a**,**c**,**e**,**f**) and grain size statistics (**b**,**d**,**f**,**h**) of (**a**,**b**) 2P; (**c**,**d**) 2P + A; (**e**,**f**) 4P; and (**g**,**h**) 4P + A samples.

Figure 3 depicts the EBSD inverse-pole figures (IPF) and corresponding grain size distributions of the 2P, 2P + A, 4P, and 4P + A samples. The 2P and 2P + A samples displayed a nonuniform structure; the grains were composed of fine grains and coarse grains. The average grain size (AGS) was about 0.71 μm after two passes. After annealing, although the proportion of fine grains decreased, the AGS remained 0.71 μm for the 2P + A sample. The 4P sample possesses a uniform grain structure and the grains were refined to about 0.42 μm. Annealing treatment resulted in a decrease in the proportion of fine grains and an increase in the AGS to about 0.43 μm for the 4P + A sample. The EBSD analysis confirmed very little grain growth during the annealing treatment.

**Figure 4:**
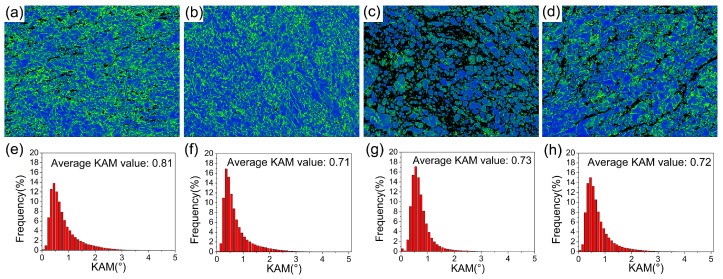
KAM maps of the (**a**) 2P, (**b**) 2P + A, (**c**) 4P, and (**d**) 4P + A samples. (**e**–**h**) The corresponding local misorientation statistics, respectively.

Figure 4 shows the EBSD kernel average misorientation (KAM) maps and the corresponding data of the 2P, 2P + A, 4P, and 4P + A samples. The dislocation density can be determined by KAM values according to the following formula [43]:

$$\rho =\frac{2\theta }{ub}$$

where ρ is dislocation density; θ stands for the average KAM value, and the θ values are presented in Figure 4; u refers to the step size in the EBSD observation; and b denotes the Burgers vector, which is 0.286 nm for pure Ti. According to the above formula, the KAM value can indicate the dislocation density. The larger KAM value implies higher dislocation density. The step size, namely, the u, is 0.09 μm for 2P and 2P + A samples, and 0.045 μm for 4P and 4P + A samples. Thus, the dislocation density in the 2P sample was ${6.3\times 10}^{16}\;m^{-2}$, and it decreased to ${5.5\times 10}^{16}\;m^{-2}$ after short-term annealing. The dislocation density in the 4P sample was the highest (${1.1\times 10}^{17}\;m^{-2}$), and it dropped a little after annealing.

**Figure 5:**
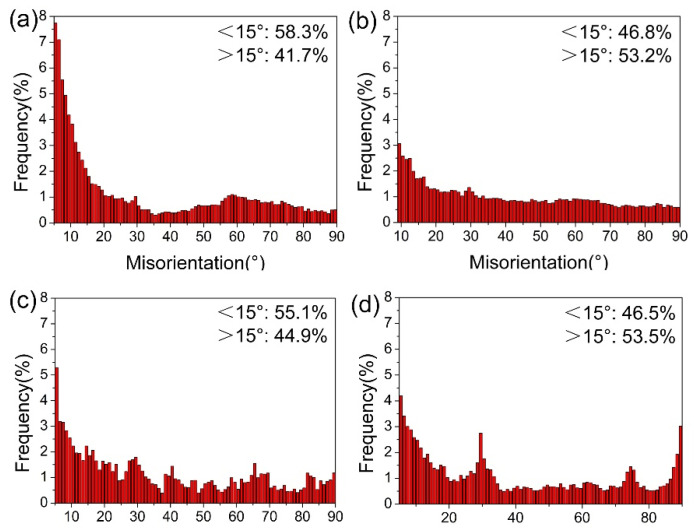
Misorientation angle distributions of (**a**) 2P, (**b**) 2P + A, (**c**) 4P, and (**d**) 4P + A samples.

**Figure 6:**
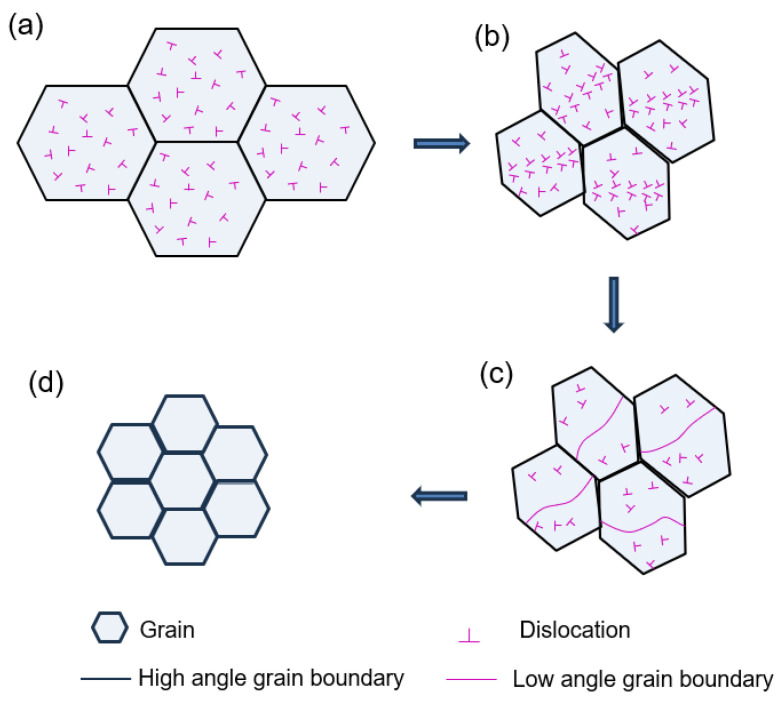
The refining mechanism pathway during ECAP. (**a**) Dislocation formation, (**b**) Dislocation rearrangement, (**c**) Formation of low angle grain boundaries, (**d**) Fine grains with high angle grain boundaries.

Figure 5 shows the misorientation angle distributions of the 2P, 2P + A, 4P, and 4P + A samples. It is considered that high-angle grain boundaries (HAGBs) refer to grain boundaries where the misorientation between adjacent grains is greater than 15°, and the rest are low-angle grain boundaries (LAGBs). The HAGBs accounted for 41.7% in the 2P sample and the proportion increased to 53.2% after annealing. In the 4P sample, the ratio of HAGBs was higher (44.9%) than that in the 2P sample. After annealing, the percentage of HAGBs increased to 53.5%. The evolution of grain boundaries for 2 and 4 passes illustrated that increasing the pass number of ECAP promoted grain refinement, accompanied by the increase in the grain boundary angle. The grain refinement mechanism pathway of 1–4 passes during ECAP is as illustrated in Figure 6. Dring ECAP, in 1–2 passes, the grains were elongated from equiaxed crystals, and the dislocation accelerated as the pass number increased. As deformation continues, dislocations accumulated and rearranged, forming structures such as dislocation walls, which further evolved into LAGBs. Then, the LAGBs partially transformed into HAGBs and divided the grains into units, thereby achieving grain refinement.

### 3.2. Texture Evolution

**Figure 7:**
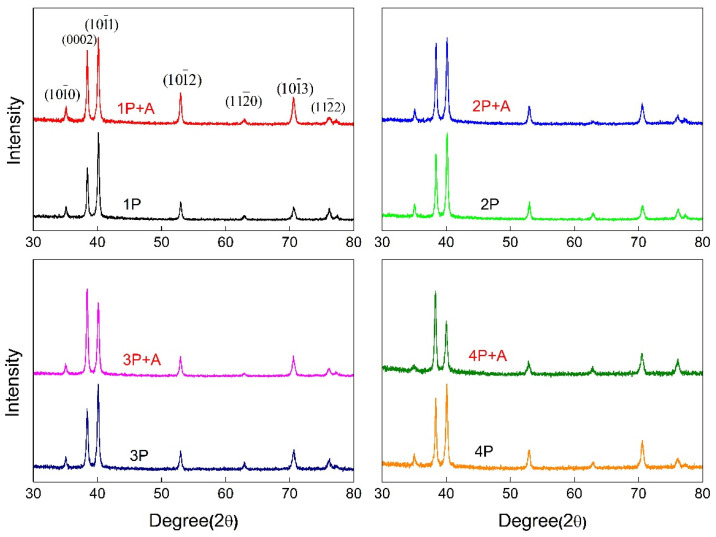
X-ray diffraction patterns of pure titanium after different processing.

Figure 7 depicts the X-ray diffraction (XRD) patterns of industrial pure titanium after ECAP processing and low-temperature short-term annealing treatment for each pass. The strongest peaks of the specimens processed via ECAP for 1–4 passes are the diffraction peaks corresponding to $\left(10\bar{1}1\right)$ crystal plane. As the number of pressing passes increased, the integrated intensity of (0002) peaks progressively increased with accumulated deformation. After low-temperature short-duration annealing treatment, the intensity of the diffraction peaks corresponding to the (0002) plane increased compared with the pre-annealing state. The strongest peak observed was the (0002) peak for the samples subjected to 3-pass or 4-pass pressing followed by annealing treatment.

**Figure 8:**
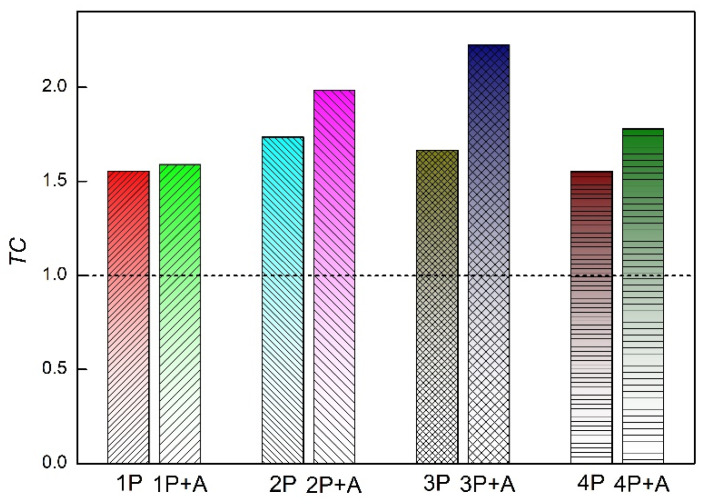
Texture coefficient of (0002) crystal planes of pure titanium after different processing.

The texture coefficient (TC) was adopted to better evaluate the preferred orientation of the grains and analyze the evolution of the texture. The texture coefficient was calculated according to Equation (1) [44,45]:

(1) $${TC}_{\left(hkil\right)}=\frac{I_{\left(hkil\right)}}{I_{0\left(hkil\right)}}{\left[\frac{1}{n}\sum_{n=1}^{n}\frac{I_{\left(hkil\right)}}{I_{0\left(hkil\right)}}\right]}^{-1}$$

where $I_{\left(hkil\right)}$ and $I_{0\left(hkil\right)}$ represent the experimentally measured diffraction intensity of the crystal plane and the standard diffraction intensity in the PDF card, respectively, and n is the number of diffraction planes. The TC value represents the degree of preferred grain orientation. A TC value of >1 indicates that grains have developed a preferred orientation. A higher TC value indicates a more pronounced preferred orientation. Based on Equation (1), the relative texture coefficients of the five crystal planes of industrial pure titanium in each state were computed; these coefficients are listed in Table 3. The texture coefficient of the (0002) crystal plane of industrial pure titanium after various processing steps is shown in Figure 8. As shown in Table 3 and Figure 8, the texture coefficients for the (0002) crystalline planes of the specimens processed via ECAP in each pass exceed 1, indicating a preferred orientation of the (0002) crystal planes parallel to sample surface. After low-temperature short-duration annealing treatment, the texture coefficients for the (0002) planes of the specimens across all passes increased, indicating a more pronounced selective orientation. The annealing process resulted in an increased alignment of the (0002) planes parallel to the specimen surfaces. The pole figures in our previous work also confirmed that the (0002) texture in 4P sample was strengthened after short-term annealing [41].

### 3.3. Mechanical Properties

**Figure 9:**
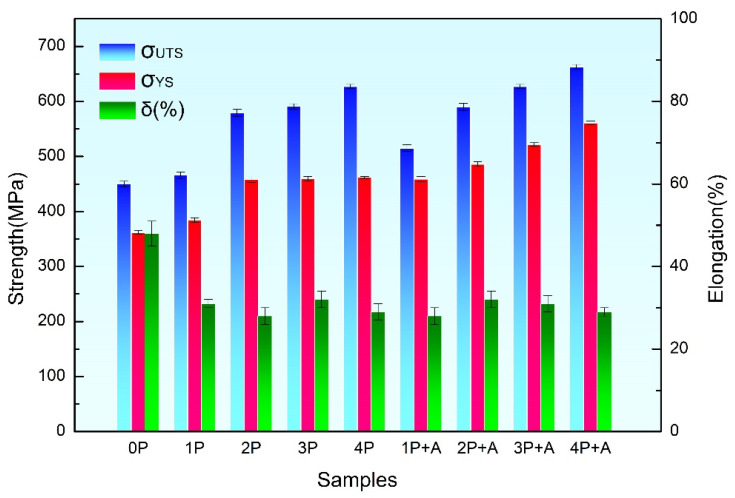
Tensile strengths, yield strengths, and elongations of pure titanium processed by ECAP for different passes and annealing.

Figure 9 and Table 4 illustrate tensile strengths, yield strengths, and elongations of pure titanium processed by ECAP for different passes and annealing. The initial tensile strength and elongation of the specimen was 450 MPa and 48%, respectively. After ECAP, the tensile strengths and yield strengths increased gradually as the pass number increased from 1 to 4. In particular, the tensile strength of specimens subjected to four passes increased to 627 MPa, reflecting a 39% improvement compared with the initial counterpart. The tensile strength of 627 MPa was close to the reported value (~630 MPa) of pure Ti after four passes of ECAP [46]. The EBSD observations proved that the grain size of the 4P sample was smaller than that of 2P sample, so the 4P sample exhibited a higher strength than the 2P sample, arising from grain refinement. Our previous work found that after 8 passes, there was almost no further improvement in strength compared to the 4-pass counterpart, because there was a dynamic balance between grain refinement and recovery [47]. Therefore, the mechanical properties after 1–4 passes were tested in the present study. The elongations of samples decreased to about 30% after ECAP. In addition, the strength of the specimens in each pass after low-temperature short-duration annealing exhibited an enhancement relative to that in their pre-annealing state. As the number of pass increased, the strengths of the annealed samples increased progressively. The tensile strength of the specimen processed through four passes and subjected to annealing treatment reached 663 MPa, indicating a 5% increase compared with its strength in the ECAP-processed condition before annealing. The annealing strengthening level of 5% was similar to the reported work [13,34]. Regarding elongation after annealing, in general, the elongation can increase after an annealing treatment. However, there were also investigations found that strength was increased and elongation was decreased after annealing in UFG Al [48,49,50], and this phenomenon was attributed to dislocation source-limited strengthening. In our present study, the elongations of annealed samples remained almost unchanged compared to their pre-annealing state. The specific underlying reasons are not clear and still need further in-depth exploration. This is the limitation of this study. We will try to address this issue in our future research.

Industrial pure titanium subjected to multiple ECAP passes after low-temperature short-duration annealing treatment exhibited a strength improvement similar to that observed for ultrafine-grained pure Cu, Al, and Mg [51,52,53]. Typically, the annealing of processed materials is beneficial for recovery and recrystallization, which results in a reduction in the defect density and an increase in the grain size, thereby decreasing the final strength. However, research has shown that applying annealing treatment to ultrafine and nanocrystalline crystals can enhance their strength, which is contrary to the conventional softening effect observed in coarse crystalline materials during annealing. Annealing strengthening exhibits different behaviors in various metals, resulting in considerable differences in the mechanistic explanations [54]. For instance, Akbari et al. [51] observed that the tensile strength of pure Cu processed increased after low-temperature short-duration annealing due to the generation of small grains during the annealing process. Zhao et al. [52] conducted annealing treatment on ultrafine crystalline aluminum at 60 °C for 30 min and ascribed the improved strength to the pinning of dislocations by the grain boundaries and the obstruction of the dislocation motion. Furthermore, Volkov et al. [53] demonstrated that the increased strength of ultrafine pure magnesium following low-temperature annealing was due to the self-locking of dislocations. Chen et al. [33] reported the unusual annealing strengthening observed in the Cu-Zn alloy and attributed this phenomenon to the impurity segregation. Semenova et al. [37] discovered that the remarkable enhancement in the strength of UFG Ti (Grade 4) after annealing was in connection with the recovery of nonequilibrium boundaries and impurity segregation.

Although various researchers have provided different explanations for the annealing strengthening phenomenon, relatively few studies have examined the influence of annealing on the texture of ultrafine crystalline materials. Compared with the cubic structure, industrial pure titanium, which has fewer slip systems, is more susceptible to texture-strengthening effects [41,55,56,57]. Texture strengthening in Al and Mg alloys has also been reported [58,59,60]. The impurity content in the industrial pure titanium used in this study was considerably low, indicating that the possibility of strengthening due to impurity segregation was substantially minimal. In this study, microstructure observation validated that the grain sizes of the annealed samples were similar to those observed before annealing treatment, indicating that the possibility of fine-grain strengthening resulting from annealing was also considerably low. The literature reported that the bimodal grain size distribution can provide additional strengthening through the hetero-deformation-induced strengthening [61]. The grain size distribution was unimodal before and after annealing in the present study, so the possibility of an extra strengthening effect by grain size distribution was slim. Based on the XRD results and texture coefficient values, the texture of the basal (0002) plane of industrial pure titanium was enhanced after annealing, which resulted in strength enhancement. In summary, the improved strength of ECAP-processed industrial pure titanium may be attributed to the following factors: (1) the low annealing temperature and short duration prevented considerable grain growth; (2) low-temperature short-duration annealing enhanced the (0002) basal texture, resulting in a small Schmid factor for the basal plane, thereby impeding dislocation movement and leading to texture strengthening, which contributed to the increased strength.

## 4. Conclusions

This study systematically investigated the microstructure and mechanical properties of industrial pure titanium subjected to 1–4 ECAP passes, followed by low-temperature short-term annealing after each pass. The results suggested a new strategy of ECAP combined low-temperature short-term annealing aimed at further increasing the strength of UFG pure titanium. In addition, this study innovatively proposed the mechanism of texture strengthening caused by annealing in UFG pure titanium. The conclusions are as follows:

(1) After ECAP processing, the microstructure of industrial pure titanium became increasingly refined with the increasing number of passes, resulting in an average grain size of ~420 nm after four passes. Furthermore, the strength of industrial pure titanium was enhanced via ECAP processing, and the tensile strength of the specimens after four passes was improved by 39% compared with that of the sample in the initial state.
(2) Microstructure observation revealed that the grain growth of ECAP-processed samples was very little during short-term annealing. The calculated results of the EBSD analysis demonstrated that the dislocation density was decreased after annealing. Moreover, the proportion of HAGBs was increased by the aging treatment. The XRD observations showed that the basal texture was strengthened after short-term annealing.
(3) The ECAP-processed specimens subjected to annealing treatment at 300 °C for 15 min exhibited increased strength compared with the pre-annealed specimens. This strengthening effect was likely attributable to the fact that the low annealing temperature and short duration prevented substantial grain growth and enhanced basal texture, thereby impeding dislocation movement.

## Figures and Tables

**Table 1:** Chemical composition of TA2 pure Ti (wt.%).

Fe	C	N	O	Ti
0.06	0.02	0.01	0.06	Balance

**Table 2:** Corresponding numbers and specific processes of different states.

Number	Specific Treatment
0P	As-received
1P	ECAP for 1 pass
2P	ECAP for 2 passes
3P	ECAP for 3 passes
4P	ECAP for 4 passes
1P + A	ECAP for 1 pass + 300 °C × 15 min annealing treatment
2P + A	ECAP for 2 passes + 300 °C × 15 min annealing treatment
3P + A	ECAP for 3 passes + 300 °C × 15 min annealing treatment
4P + A	ECAP for 4 passes + 300 °C × 15 min annealing treatment

**Table 3:** Texture coefficients of pure titanium after different processes.

Sample	Texture Coefficient (*TC*)				
	$\left(10\bar{1}0\right)$	(0002)	$\left(10\bar{1}1\right)$	$\left(10\bar{1}2\right)$	$\left(11\bar{2}0\right)$
1P	0.52	1.55	0.76	1.29	0.49
2P	0.55	1.73	0.69	1.17	0.63
3P	0.52	1.66	0.68	1.31	0.68
4P	0.49	1.55	0.55	1.11	0.60
1P + A	0.51	1.59	0.55	1.62	0.45
2P + A	0.51	1.98	0.63	1.07	0.34
3P + A	0.43	2.22	0.56	1.23	0.30
4P + A	0.36	1.78	0.35	0.90	0.73

**Table 4:** Corresponding data of tensile strengths, yield strengths, and elongations of pure titanium in each state.

Samples	Tensile Strength σb (MPa)	Yield Strength σs (MPa)	Elongations δ (%)
0P	450 ± 5	362 ± 3	48 ± 3
1P	466 ± 6	384 ± 4	31 ± 1
2P	579 ± 7	458 ± 5	28 ± 2
3P	591 ± 5	460 ± 4	32 ± 2
4P	627 ± 4	462 ± 3	29 ± 2
1P + A1	515 ± 6	459 ± 5	28 ± 2
2P + A1	590 ± 7	486 ± 4	32 ± 2
3P + A1	627 ± 4	522 ± 3	31 ± 2
4P + A1	663 ± 4	561 ± 3	29 ± 1

## Data Availability

The original contributions presented in this study are included in the article. Further inquiries can be directed to the corresponding author.
